# 2100. Epidemiology and Risk Factors for Invasive Fungal Infections Among Patients with Hematological Malignancies In Colombia

**DOI:** 10.1093/ofid/ofac492.1722

**Published:** 2022-12-15

**Authors:** Yeimer Ortiz-Martínez, Javier Fajardo-Rivero, Tania Mendoza-Herrera, Claudia Figueroa-Pineda, Carlos Ruiz-González

**Affiliations:** Universidad Industrial de Santander, Bucaramanga, Santander, Colombia; Universidad Industrial de Santander, Bucaramanga, Santander, Colombia; Universidad Industrial de Santander, Bucaramanga, Santander, Colombia; Universidad Industrial de Santander, Bucaramanga, Santander, Colombia; Universidad Industrial de Santander, Bucaramanga, Santander, Colombia

## Abstract

**Background:**

Invasive fungal infection (IFI) is a potentially lethal complication in patients with hematological malignancies (HM). However, studies are scarce in this population, especially in developing countries. The aim of this study was to investigate the prevalence, epidemiology, predictive factors, and outcomes of IFI in patients with HM hospitalized in non-HEPA-filtered rooms (resource-limited settings) in a reference center in Colombia.

**Methods:**

A cross-sectional, retrospective study was conducted on the clinical data of HM patients and pulmonary infection hospitalized in a tertiary university hospital in Bucaramanga, Colombia, between 2015 and 2020. The primary outcome was proven/probable IFI according to the EORTC/MSGERC criteria. A descriptive and group comparison analysis were performed between patients with IFI and patients with non-fungal infections. The main risk factors for the development of IFI were identified by multivariate stepwise logistic regression analysis.

**Table 1:**
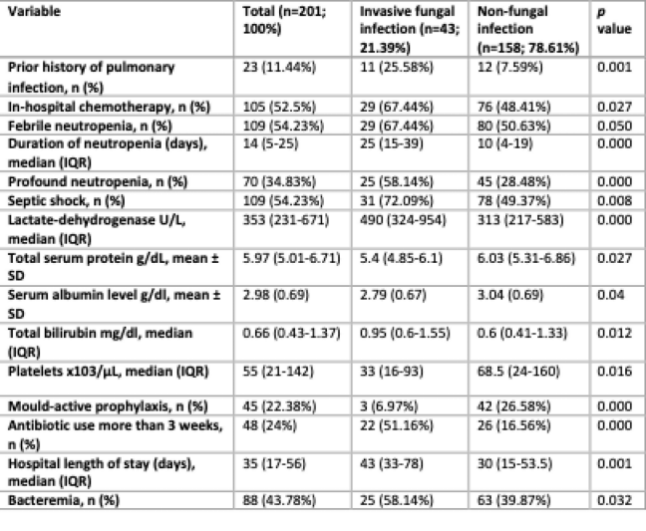
Statistically significant variables in the univariate analysis of patient characteristics compared between patients with invasive fungal infection and patients with non-fungal infection.

**Image 1:**
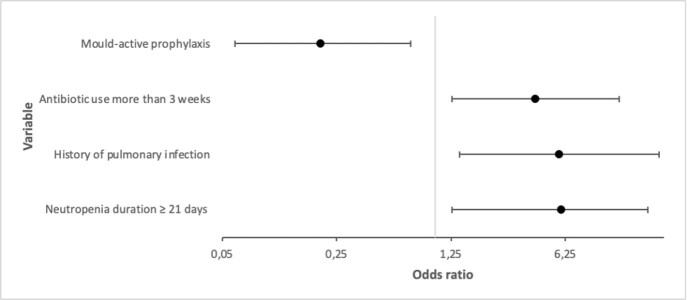
Associated factors to develop invasive fungal infections (IFIs) in patients with HM. The graph shows the variables showed statistically significant difference in the multivariate analysis.

**Results:**

In 201 patients, the prevalence of proven/probable IFI was 21.39% (43 cases). The most common IFI was caused by *Aspergillus spp.* (41.8%), followed by *Candida spp.* (34.8%), *Mucor spp.* (6.9%), *Penicillium spp*. (4.6%) and *Cryptococcus neoformans* (4.6%). The lung was the most commonly affected site (n=34; 81.3%); four patients (9.3%) developed fungal sinusitis and disseminated IFI, respectively. In-hospital mortality was 86% (37/43). Multivariate logistic regression analysis (area under the ROC curve=0.90) showed that antibiotic use ≥3 weeks (OR, 4.11; 95% CI, 1.26-13.31; *p*< .001), history of pulmonary infection (OR, 5.75; 95% CI, 1.41-23.39; *p*< .001) and neutropenia duration ≥21 days (OR, 5.88; 95% CI, 1.26-19.86; *p* < .001) were independent risk factors for the development of IFI and mould-active prophylaxis (OR: 0.20; 95% CI: 0.060–0.705; *p*=0.012) was significantly associated with a lower occurrence of IFI.

**Conclusion:**

HM patients in resource-limited settings have a high prevalence of IFI with an elevated mortality. The use of mould-active prophylaxis is associated with a significantly lower occurrence of IFI. Cost-effective strategies for prevention and early diagnosis of IFI are required to improve survival in patients with HM.

**Disclosures:**

**All Authors**: No reported disclosures.

